# Humidity Measurement in Carbon Dioxide with Capacitive Humidity Sensors at Low Temperature and Pressure

**DOI:** 10.3390/s18082615

**Published:** 2018-08-09

**Authors:** Andreas Lorek, Jacek Majewski

**Affiliations:** 1 German Aerospace Center (DLR), Rutherfordstraße 2, 12489 Berlin, Germany; 2 Department of Automation and Metrology, Faculty of Electrical Engineering and Computer Science, Lublin University of Technology, 38A Nadbystrzycka Str., 20-618 Lublin, Poland; j.majewski@pollub.pl

**Keywords:** capacitive humidity sensors, SHT75, carbon dioxide, humidity, Mars in-situ measurements, experimental simulation chambers, Martian atmosphere, low temperature, low pressure, CO_2_

## Abstract

In experimental chambers for simulating the atmospheric near-surface conditions of Mars, or in situ measurements on Mars, the measurement of the humidity in carbon dioxide gas at low temperature and under low pressure is needed. For this purpose, polymer-based capacitive humidity sensors are used; however, these sensors are designed for measuring the humidity in the air on the Earth. The manufacturers provide only the generic calibration equation for standard environmental conditions in air, and temperature corrections of humidity signal. Because of the lack of freely available information regarding the behavior of the sensors in CO_2_, the range of reliable results is limited. For these reasons, capacitive humidity sensors (Sensirion SHT75) were tested at the German Aerospace Center (DLR) in its Martian Simulation Facility (MSF). The sensors were investigated in cells with a continuously humidified carbon dioxide flow, for temperatures between −70 °C and 10 °C, and pressures between 10 hPa and 1000 hPa. For 28 temperature–pressure combinations, the sensor calibration equations were calculated together with temperature–dependent formulas for the coefficients of the equations. The characteristic curves obtained from the tests in CO_2_ and in air were compared for selected temperature–pressure combinations. The results document a strong cross-sensitivity of the sensors to CO_2_ and, compared with air, a strong pressure sensitivity as well. The reason could be an interaction of the molecules of CO_2_ with the adsorption sites on the thin polymeric sensing layer. In these circumstances, an individual calibration for each pressure with respect to temperature is required. The performed experiments have shown that this kind of sensor can be a suitable, lightweight, and relatively inexpensive choice for applications in harsh environments such as on Mars.

## 1. Introduction

The exploration of Mars has become of growing importance in view of its relatively promising Martian environmental conditions for extraterrestrial forms of life [1,2]. One of the main goals of Mars investigation is “to follow the water” [3] as a prerequisite for the survival of living entities. Because of low temperatures (e.g., 215 K to 273 K at the equator) [3] and low Martian atmospheric pressure (600–800 Pa near the surface) [1], water can exist only as vapor, as ice, in brines [4], or bounded on the surface of the regolith as interfacial water in a liquid-like state [5], and it might also form by the process of deliquescence [6]. The water vapor is of particular interest, because it influences chemical reactions; because of the water content in the lower atmosphere and upper regolith, the phenomena like fog and thin frost layers occur, and could also be important for potential life forms. The question of water vapor content in the Martian atmosphere, with a dominant part of 96% carbon dioxide [7], can be resolved by accurate measurements of the relative humidity (*U*_w,i_) in carbon dioxide at low temperatures and pressures. It is important to determine the metrological properties of humidity sensors in this extraterrestrial environment, prior to mounting onto a lander or rover mission or to use in Mars-simulating chambers.

This paper presents the results of the investigation on the SHT75 relative humidity sensors (Sensirion, Steafa ZH, Switzerland) in CO_2_, performed at the DLR (German Aerospace Center) laboratory in Berlin. A similar investigation in the regular air of the Earth, performed in the same laboratory, has already been described in the literature [8]. That former paper [6] is essential for the understanding of the present paper, because the experimental setup, definitions of parameters, and so on, that are used in the present paper, have been explained and defined in the literature [8].

The SHT75 sensor is of polymer-based capacitive type, and both in the Mars simulation chambers on Earth and on the Martian rovers, that type of sensor is generally applied. For example, in the MESCH chamber (Mars Environmental Simulation Chamber) developed at the University of Aarhus (Aarhus C, Denmark), the Honeywell sensor HIH-3602C (Honeywell International Inc., Golden Valley, MN, USA) was used [9], and in the MARTE chamber (Mars environmental simulation chamber) built at Centro de Astrobiologia in Madrid, the Honeywell sensor HIH-4000 (Honeywell International Inc., Golden Valley, MN, USA) was employed [10]. In the PELS (Planetary Environmental Liquid Simulator) system at the University of Edinburgh, the Honeywell sensor HIH-4602-A (Honeywell International Inc., Golden Valley, MN, USA) was applied [11]. In the Phoenix spacecraft that landed on Mars in 2008, its instrument, MECA (Microscopy, Electrochemistry, and Conductivity Analyzer), contained a probe TECP (Thermal and Electrical Conductivity Probe) with the Panametrics sensor MiniCap-2 (GE Panametrics, Waltham, MA, USA) [12]. Most recently, the Curiosity rover, operating on Mars from 2012, contains the REMS instruments (Rover Environmental Monitoring Station) set with three Humicap sensors (Vaisala, Helsinki, Finland) [13]. The choice of polymer-based capacitance sensors is based on a number of advantages such as small dimensions and lightweight, low energy consumption, simple electronics for sensor’s signal conditioning, a reliable measurement principle providing linear characteristics, and a relatively short response time. Sensirion AG belongs to the worldwide market-leading manufacturers of the humidity sensors, and the SHT75 sensor design exhibits high metrological properties.

## 2. Experimental Procedure

In the case of measurements in carbon dioxide, the number of measuring points (each point collected at stable pressure, temperature, humidity, and stable output signals of the reference dew point hygrometer and the investigated SHT75 sensors) taken into account (1316) was considerably smaller than for the measurements in air (5244), carried out in similar experiments at the DLR in 2013 [8]. Seven of the nine SHT75 sensors used in the air experiments were afterward tested in the carbon dioxide experiments.

The developed gas mixing system can generate either humidified air containing the amounts of water vapor that correspond to the humidity levels occurring in the atmospheric air on Earth, or the gas compositions corresponding to the atmosphere at the surface of Mars. The essential parts of the system are the adjustable mass flow controllers. As up to six individual gas components can be blended in the system, one controller per each inlet (including the control of the gas stream to be humidified) is used, and one controller per each of three outlets leading to the three measuring cells; nine controllers in total. In each measuring cell, three SHT75 sensors are placed. At 1013.25 hPa, the system can generate dew/frost points ranging from −82 °C dry air frost point (*t*_f_) to 5 °C dew point (*t*_d_).

In place of the dry air that is used in the investigation described in the literature [8], in the experiments discussed here, CO_2_ gas delivered in bottles was used as the carrier gas, with the purity of 99.995% and the frost point of −66 °C. The system can provide a continuous flow of a carrier gas up to 150 L/h at 20 °C and 1000 hPa [14]. A part of the dry carrier gas stream is saturated with water vapor when bubbled through liquid water in the scrubber bottles placed in the thermostat regulated water bath. That method ensures that a stable frost point temperature *t*_f_ of the humidified carrier gas with the setting accuracy of ±0.5 °C is maintained. When decompressing the humidified gas inside the measuring cells to 10 hPa with a vacuum pump, the dew point range of −94 to −46 °C can be reached. Only the results obtained from seven of the nine investigated sensors were considered to be valid for analyzing, because at the end of the experiments, the two sensors excluded from the analysis exhibited excessive deviations at the relative humidity above 80%.

The measurements in carbon dioxide were made in the temperature range of 10 °C to −70 °C in 10 K steps, in monotonically decreasing order (nine temperature steps altogether). In each temperature step, the pressure was monotonically decreased. Firstly, the measurements at 1000 hPa were performed, and then the pressure was decreased down to 500 hPa, 200 hPa, and (from −30 °C downwards) to 10 hPa. Within every pressure step, firstly, the humidity was decreased in steps, and then increased back. For any combination of temperature and pressure values (i.e., of row and column headings of Table 1), the corresponding set of measurement points was taken only once.

In Table 1, the applied ranges of the relative humidity for every temperature–pressure combination are listed. The measurement points for 10 hPa and 200 hPa at 10 °C and for 10 hPa at 0 °C, −10 °C, and −20 °C could not be obtained because of the limitation of the gas mixing system. The measurement points at −70 °C for 500 hPa and 1000 hPa were not taken as a result of very long response times of the SHT75 sensors (tens of hours at higher pressure values).

## 3. Results

### 3.1. Pressure Dependency of the SHT75 in CO_2_

**Figure 1:**
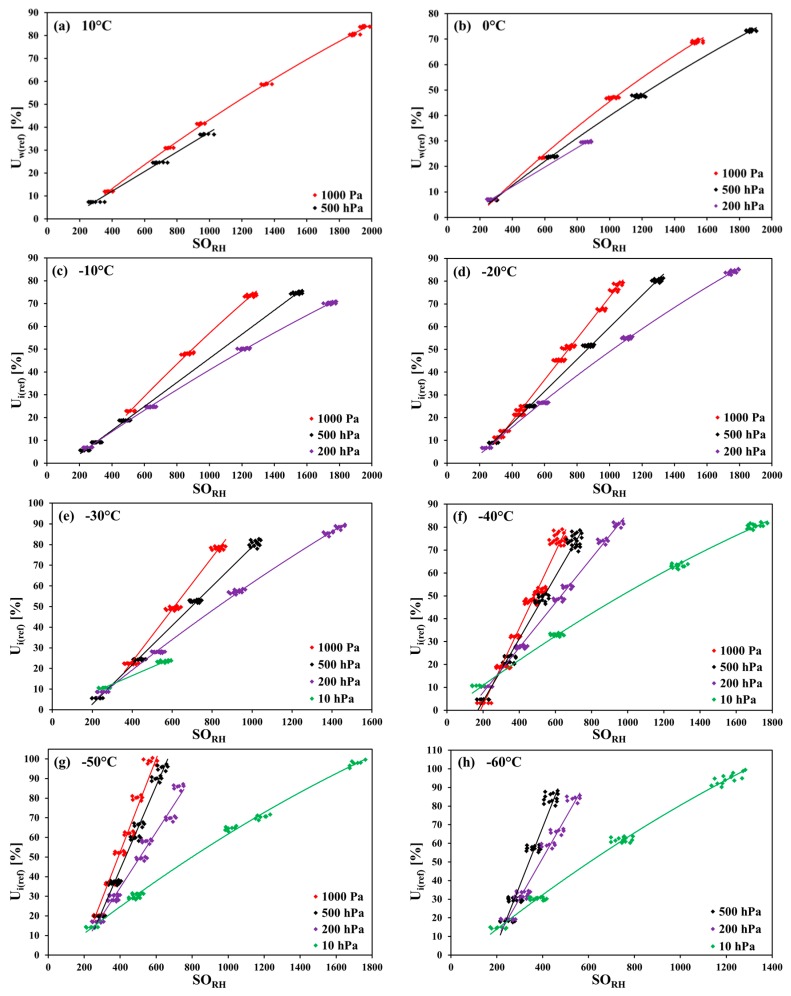

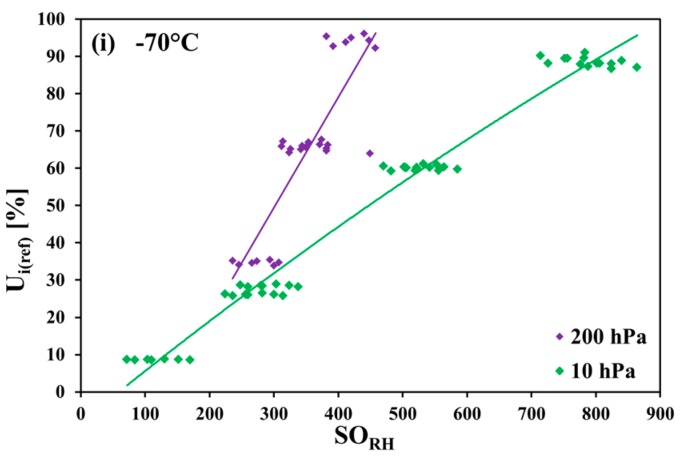
Pressure dependencies of the SHT75 sensors in CO_2_ (regression lines fitted to measurement points collected from all tested sensors at different temperature/pressure combinations) at temperatures from −70 °C to 10 °C; SO_RH_ are the integer rough values (SO means ‘sensor output’, i.e., the humidity readout) of the SHT75 sensors.

Figure 1a–i show the pressure dependency of all of the SHT75 sensors investigated in CO_2_ at various temperatures. In Figure 1e–g, each of the results of the fits at four different pressures are plotted. The reasons for the lack of one (Figure 1b–d,h) or two (Figure 1a,i) fit lines are explained above (comment on Table 1), or the slope of the fit line was too steep so that the resolution of the humidity readout strongly decreased, and an analysis has made no sense.

The pressure has a significant influence on the measured values of humidity, especially at lower pressure ranges. The slopes at 1000 hPa are always the greatest ones, whereas at 10 hPa (or if absent, at 200 hPa), the fitted lines have the least steep slopes. This pressure influence becomes more conspicuous with the temperature falling. At −30 °C, the value for the slope of the 1000 hPa fit line is only three times greater than that of the 10 hPa line, while at −50 °C the ratio is four to one.

### 3.2. Temperature Dependency of the SHT75 in CO_2_

**Figure 2:**
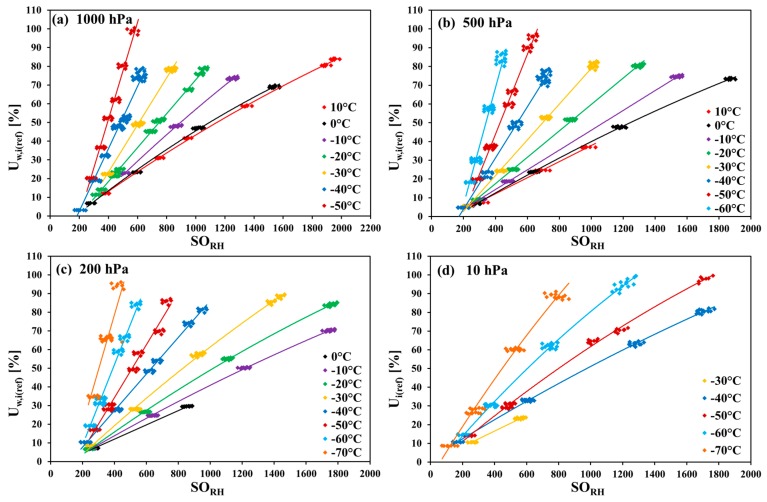
Temperature dependencies of the SHT75 sensors in CO_2_ (regression lines fitted to measurement points collected from all tested sensors at different temperature-pressure combinations) at pressures from 10 hPa to 1000 hPa.

The set of fits depicted in Figure 1a–i (a separate figure for each constant temperature value) can be rearranged and divided into four other figures (a separate figure for each constant pressure value). Figure 2a–d show the temperature dependency of the fits at different pressures.

The four figures above show that the slope of the fit becomes greater with the decreasing temperature. The slope values at 10 °C and 0 °C are similar for 500 hPa and 1000 hPa. The difference increases at −10 °C and grows with decreasing temperature. For 1000 hPa, the slope value at −50 °C is four times greater than that at 10 °C. On the other hand, for 10 hPa, the slope value at −70 °C is only ca. three times greater than at −30 °C. The fit equations are listed in Table 2.

The equations in Table 2 are written in the following form:

(1) $$U_{w,i\left(\mathrm{ref}\right)}=a_{2}\times SO_{RH}{}^{2}+a_{1}\times SO_{RH}+a_{0}\;\mathrm{for}\;\mathrm{polynomial}\;\left(\mathrm{quadratic}\right)\;\mathrm{regression}\;\mathrm{fits},$$

(2) $$U_{w,i\left(\mathrm{ref}\right)}=a_{1}\times SO_{RH}+a_{0}\;\mathrm{for}\;\mathrm{linear}\;\mathrm{regression}\;\mathrm{fits}.$$

For each investigated pressure value, an individual regression equation is needed with its own slope and intercept value.

**Figure 3:**
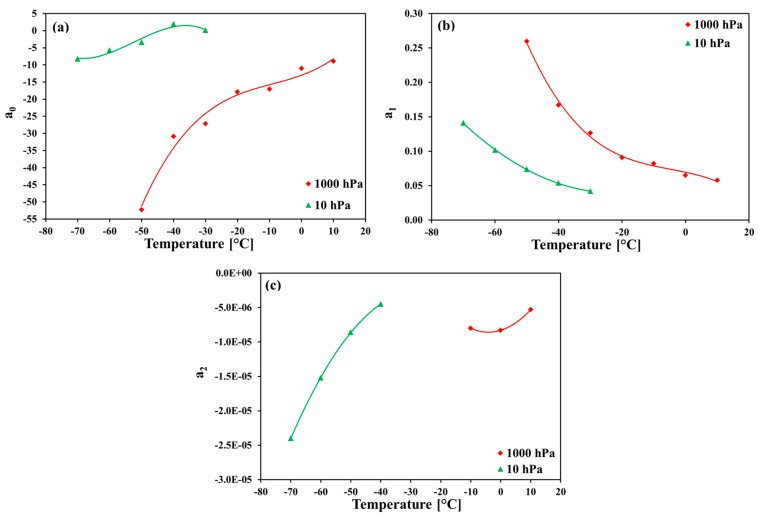
Temperature dependencies of the parameters *a*_0_, *a*_1_, and *a*_2_ at pressures 1000 hPa and 10 hPa (on the basis of the values from Table 2).

For exemplification, the relationship between temperature and the parameters *a*_0_, *a*_1_, and *a*_2_ from Equations (1) and (2), is plotted for the pressures 1000 hPa and 10 hPa in Figure 3a–c (on the basis of the values from Table 2).

The polynomial regression fit equations of the temperature dependencies of the parameters *a*_0_, *a*_1_, and *a*_2_ are listed in Table 3 (*t* denotes the sensor temperature).

### 3.3. Cross-Sensitivity of the SHT75 to CO_2_

**Figure 4:**
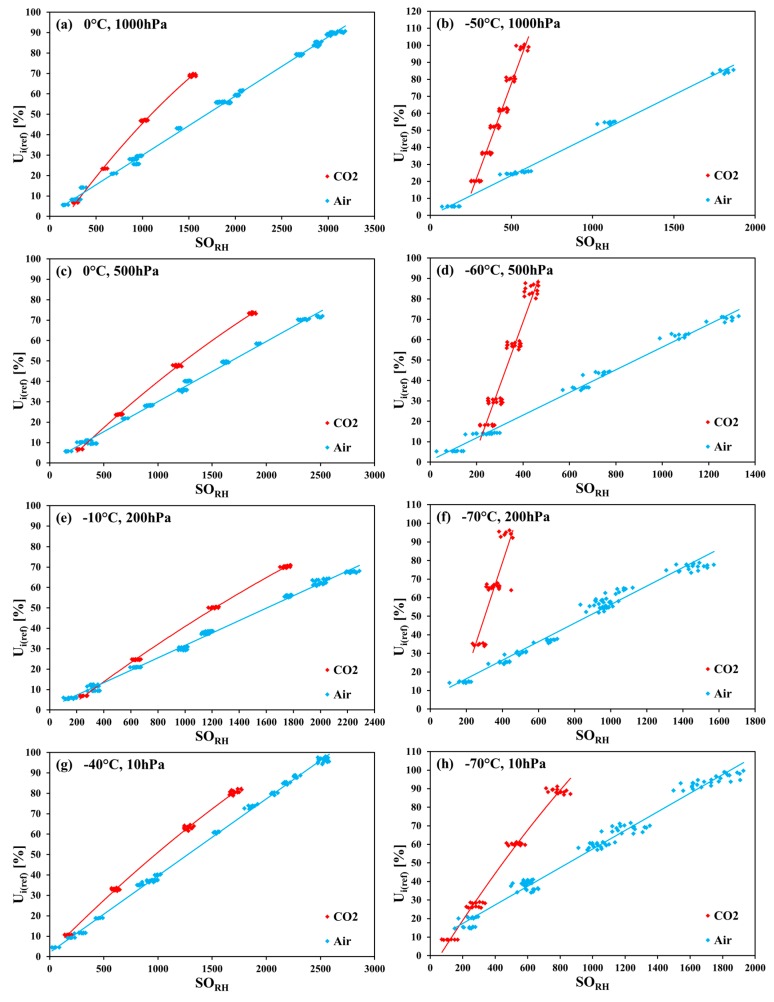
Exemplary collations of the SHT75 sensor characteristic curves (regression lines fitted to measurement points collected from all tested sensors) for the relative humidity measurement in CO_2_ and in air, at selected temperature–pressure combinations.

The substitution of the air with CO_2_ as the carrier gas has led to some serious and unanticipated consequences. In exemplary Figure 4a–h, the characteristic curves based on the relative humidity values measured in the CO_2_ atmosphere are compared with the curves based on the values measured in the air (taken from [8]), for the same selected temperature–pressure combinations. A conspicuous cross-sensitivity for any pressure and temperature is observed, with a strong increase at significantly decreased temperatures. For a given humidity value, this cross-sensitivity results in lowered *SO_RH_* values being obtained in CO_2_ and also in lower resolution, and a higher uncertainty of the measured values when compared with those obtained in the air.

In Figure 4a–h, eight pairs of linear (or slightly quadratic) characteristics are shown. Each pair consists of one line for a set of measurement points collected with the SHT75 sensors in air, and one line obtained in CO_2_. The first related pair of figures (Figure 4a,b) are collated for 1000 hPa, and the following pairs are below—for 500 hPa, 200 hPa, and 10 hPa (Figure 4g,h). In each pair of figures, the fits at higher and lower temperature (for the same pressure value) are compared.

In order to evaluate the strength of the cross-sensitivity, the ratio of two slopes, namely the slope of the linear characteristics obtained in CO_2_ and in air (*p* and *T* being equal), can be used. That ratio for the right-column plots (low *T*) is for pressures of 200 hPa, 500 hPa, and 1000 hPa are 4.7, 4, and 3 times greater, respectively, than for the coupled left-column plots (*T* = 0 °C or −10°C). For *p* = 10 hPa, the ratio of the slopes calculated at and −70 °C is only 1.9 times greater than at −40 °C. The comparison of Figure 4a–f (200 hPa to 1000 hPa) with Figure 4g–h (10 hPa) shows that the results of the measurements in a rarified carbon dioxide atmosphere are the most close to the measurements in air, but the best resolution is obtained at near-zero degrees Celsius temperatures.

## 4. Discussion

The measurements seem to prove a strong cross-sensitivity of the SHT75 sensors to CO_2_. What could be the reason?

Inside the measuring system (Figure 1 in [8]), three main areas of the influence of carbon dioxide on the results may be suggested.

The first area of the possible interactions of carbon dioxide and water vapor is the gas mixing system, in which carbon dioxide is humidified and then mixed with dry carbon oxide in a given proportion, which determines the relative humidity of the mixture. During the mixing process, there is a possibility that some molecules of the gaseous carbon dioxide and the water vapor react to form molecules of gaseous carbonic acid (H_2_CO_3_). Some researchers suppose that the gaseous carbonic acid is present in cirrus clouds in the Earth’s atmosphere and in the atmosphere on Mars [15]. In laboratory experiments, solid or gaseous carbonic acid is formed by the high-energy irradiation of H_2_O/CO_2_-ice or by acid–base chemistry at cryotemperatures. In the Earth’s troposphere, under low humidity and at 250 K, the slow decomposition of H_2_CO_3_ is suggested. Under ambient conditions, the molecules of carbonic acid are very unstable if contact with water molecules is possible. However, a small portion of water vapor molecules might react with carbon dioxide, thereby reducing the amount of moisture measured by the sensor. In liquid water saturated with gaseous carbon dioxide, only ca. 0.2% of CO_2_ [16] is bonded as carbonic acid; a similar proportion might be assumed in a mixture of water vapor and gaseous CO_2_.

The second area is the chilled mirror surface of the dew point hygrometer. CO_2_ or its reaction products could influence the dew point measurement. On the chilled mirror surface of the hygrometer, condensed water droplets or the deposition of frost crystals occur. The gaseous carbon dioxide is easily soluble in liquid water, especially at low temperatures, and also in the water droplets on the mirror. Then, inside the droplets, carbonic acid might form. But again, the molecules of carbonic acid inside the water droplets would be very unstable, and the droplets themselves evaporate frequently. In the case of frost, as the carbon dioxide molecules are large in comparison with water molecules in ice, the difference in kinetic diameters (H_2_O [0.265 nm] vs. CO_2_ [0.330 nm]) is unfavorable for the penetration of CO_2_ into frost crystals. CO_2_ could also freeze out on the chilled mirror surface, but the sublimation point is −78.5 °C at 1013.25 hPa [17] and the hygrometer has not reached such low temperature at 1000 hPa. Finally, the calculated values from the gas mixing system were in agreement with the values measured with the hygrometer. For these reasons, the use of a dew point mirror hygrometer should not cause the strong deviation of the SHT75 sensor measurement values in CO_2_ from the values measured in air.

The third area is the sensing layer of the sensor. A competition of water vapor molecules and CO_2_ molecules for the access to functional groups of an adsorbing surface was observed in the case of carboxyl groups on the carbon surface [18]. The polarity of water molecules is well known, but also, the carbon dioxide molecules exhibit a slight polarity. In the O=C=O molecule considered as quasi-linear, the end oxygen atoms are slightly electronegative, whereas the slight positive charge is located near the central carbon atom [19]. The polymers applied as a sensing layer in the capacitive humidity sensors are mostly polyimide-based, and the most popular are the various polyimides similar to Kapton^®^. In the Kapton^®^ structure, the carboxyl groups –C=O are the primary bonding sites for the adsorption of water vapor molecules. Also, the ether groups C–O–C and N–C groups can constitute adsorption sites [20].

The most probable explanation for a strong cross-sensitivity of SHT75 humidity sensors to carbon dioxide assumes that the molecules of CO_2_ interact with the adsorption sites on the thin polymer layer. Firstly, the CO_2_ molecules can produce weak hydrogen bonds between the O or N atoms in the polymer chains, and the carbon atom in the CO_2_ molecule. Secondly, the CO_2_ molecules can attach to water molecules that have created hydrogen bondings at primary adsorption sites. Then, no more water molecules can be adsorbed as dimers or clusters at an adsorption site blocked by CO_2_ molecules, and the amount of moisture adsorbed on the polymer sensing layer is strongly reduced. This explanation may justify the steepest slopes (and the smallest output signals) at higher pressures of the humidified CO_2_. The increase of the slope values when the temperature falls, although observed in air, is much stronger in carbon dioxide. Here, the explanation could be the difficulties in penetrating the water molecules inside the polymer layer when the thermal movements of the polymer chains are reduced, together with the presence of relatively bigger carbon dioxide molecules adsorbed on the polymer sites, which partly block the ways of penetration for much smaller water vapor molecules.

Based on the collected data, this kind of sensor seems to be a reasonable choice for application in the harsh environment on Mars. A major disadvantage is the limitation of the measurement range, down to about *U*_i_ = 5% for low humidity. That limit could be reached when the temperature of the atmosphere surrounding the sensor is above −55 °C at a frost point of *t*_fp_ = −76 °C [21]. Thus, for the measurement of the *U*_i_ or *U*_w_ values below that lower range limit of the polymer-based capacitive sensors, at higher temperatures, another sensor working principle is necessary (e.g., that of a coulometric sensor). The high *U*_i_ values above 95% can also be difficult to measure. For the measurement of the frost point (*U*_i_ = 100%), a thin plate coupled with a precise temperature measurement that allows for detecting the adsorption and desorption of condensed water on the plate, could be used. Such considerations and measurements are described in the literature [22].

## 5. Conclusions

The measurements of the relative humidity of the gaseous carbon dioxide using polymer-based capacitive humidity sensors revealed a strong dependence of the sensor characteristic curve on both the temperature and the pressure of the measured humid gas. The greatest deviation from the sensor nominal characteristic curve was observed at the lowest investigated temperature of −70 °C and at the pressures 200 hPa, 500 hPa, and 1000 hPa.

The comparison with the results obtained for the same sensors in the measurements of humid air showed big discrepancies that demonstrate the considerable cross-sensitivity of the sensors to carbon dioxide. The most probable explanation of this effect can be the interactions of carbon dioxide molecules both directly with the adsorption sites on the polymer layer, and indirectly with the water molecules adsorbed on the primary adsorption sites on the polymer.

Despite of the observed cross-sensitivity, the polymer-based capacitive sensors can still be used for the measurements of relative humidity in carbon dioxide at low pressures, within a broad range of temperatures. An individual calibration of the sensors for such applications is recommended, and earlier experiments should be checked for whether the cross-sensitivity has not been taken into account. The research on cross-sensitivity to various gases for this type of humidity sensor should be continued.

## Figures and Tables

**Table 1:** Range of the relative humidity of CO_2_ under investigation (minimum and maximum values) for different temperature and pressure conditions. The relative humidity (Equation (1) and Section 3.2 in [8]) is calculated with respect to water *U*_w_ or ice *U*_i_ (marked by brackets). *T* denotes humid gas temperature (in °C).

*T*	*p*			
	1000 hPa	500 hPa	200 hPa	10 hPa
**10 °C**	84	37	-	-
	12	7	-	-
**0 °C**	70	74	30	-
	7	7	7	-
**−10 °C**	67 (74)	68 (75)	64 (71)	-
	21 (23)	5 (6)	6 (7)	-
**−20 °C**	65 (79)	67 (81)	70 (85)	-
	9 (11)	7 (9)	5 (7)	-
**−30 °C**	59 (80)	62 (83)	67 (90)	18 (24)
	16 (22)	4 (5)	6 (8)	8 (10)
**−40 °C**	54 (79)	53 (79)	56 (83)	56 (82)
	2 (3)	3 (5)	7 (10)	7 (10)
**−50 °C**	(100)	(97)	(87)	(100)
	(20)	(19)	(17)	(14)
**−60 °C**	-	(88)	(86)	(99)
	-	(18)	(19)	(14)
**−70 °C**	-	-	(96)	(91)
	-	-	(34)	(9)

**Table 2:** Polynomial (quadratic) and linear regression fit equations for each pressure–temperature pair shown in Figure 1a–i and Figure 2a–d.

Pressure [hPa]	Temperature [°C]	Fit Equation
**1000**	10	$U_{w,i\left(\mathrm{ref}\right)}=-0.0000053\times SO_{RH}{}^{2}+0.0575737\times SO_{RH}-8.96$
	0	$U_{w,i\left(\mathrm{ref}\right)}=-0.0000083\times SO_{RH}{}^{2}+0.0648922\times SO_{RH}-11.04$
	−10	$U_{w,i\left(\mathrm{ref}\right)}=-0.000008\times SO_{RH}{}^{2}+0.0821515\times SO_{RH}-17.08$
	−20	$U_{w,i\left(\mathrm{ref}\right)}=0.0908286\times SO_{RH}-17.84$
	−30	$U_{w,i\left(\mathrm{ref}\right)}=0.1263865\times SO_{RH}-27.19$
	−40	$U_{w,i\left(\mathrm{ref}\right)}=0.1668640\times SO_{RH}-30.88$
	−50	$U_{w,i\left(\mathrm{ref}\right)}=0.2595892\times SO_{RH}-52.28$
**500**	10	$U_{w,i\left(\mathrm{ref}\right)}=0.0428904\times SO_{RH}-5.033$
	0	$U_{w,i\left(\mathrm{ref}\right)}=-0.0000049\times SO_{RH}{}^{2}+0.0523972\times SO_{RH}-7.62$
	−10	$U_{w,i\left(\mathrm{ref}\right)}=0.00000025\times SO_{RH}{}^{2}+0.05227031\times SO_{RH}-6.55$
	−20	$U_{w,i\left(\mathrm{ref}\right)}=0.0703082\times SO_{RH}-10.59$
	−30	$U_{w,i\left(\mathrm{ref}\right)}=0.0952314\times SO_{RH}-16.2$
	−40	$U_{w,i\left(\mathrm{ref}\right)}=0.1362861\times SO_{RH}-23.27$
	−50	$U_{w,i\left(\mathrm{ref}\right)}=0.212559\times SO_{RH}-41.17$
	−60	$U_{w,i\left(\mathrm{ref}\right)}=0.3125689\times SO_{RH}-56.36$
**200**	0	$U_{w,i\left(\mathrm{ref}\right)}=0.0381205\times SO_{RH}-3.13$
	−10	$U_{w,i\left(\mathrm{ref}\right)}=-0.0000043\times SO_{RH}{}^{2}+0.0510756\times SO_{RH}-5.88$
	−20	$U_{w,i\left(\mathrm{ref}\right)}=-0.0000067\times SO_{RH}{}^{2}+0.064592\times SO_{RH}-8.87$
	−30	$U_{w,i\left(\mathrm{ref}\right)}=-0.0000082\times SO_{RH}{}^{2}+0.0817805\times SO_{RH}-12.12$
	−40	$U_{w,i\left(\mathrm{ref}\right)}=0.0978043\times SO_{RH}-11.71$
	−50	$U_{w,i\left(\mathrm{ref}\right)}=0.1414872\times SO_{RH}-22.15$
	−60	$U_{w,i\left(\mathrm{ref}\right)}=0.2155412\times SO_{RH}-34.06$
	−70	$U_{w,i\left(\mathrm{ref}\right)}=0.2965787\times SO_{RH}-39.6$
**10**	−30	$U_{w,i\left(\mathrm{ref}\right)}=0.0417898\times SO_{RH}-0.11$
	−40	$U_{w,i\left(\mathrm{ref}\right)}=-0.0000045\times SO_{RH}{}^{2}+0.0537153\times SO_{RH}+1.88$
	−50	$U_{w,i\left(\mathrm{ref}\right)}=-0.0000086\times SO_{RH}{}^{2}+0.0737212\times SO_{RH}-3.41$
	−60	$U_{w,i\left(\mathrm{ref}\right)}=-0.0000152\times SO_{RH}{}^{2}+0.1013665\times SO_{RH}-5.73$
	−70	$U_{w,i\left(\mathrm{ref}\right)}=-0.000024\times SO_{RH}{}^{2}+0.1409922\times SO_{RH}-8.25$

**Table 3:** Polynomial regression fit equations of the curves shown in Figure 3a–c.

Pressure	Equation	Range of Validity
**1000 hPa**	$a_{0}=3.7153\times 10^{-4}t^{3}+0.01015t^{2}+0.34192t-13$	−50 °C to 10 °C
	$a_{1}=-1.5502\times 10^{-6}t^{3}-2.1957\times 10^{-5}t^{2}-9.836\times 10^{-4}t+0.07$	−50 °C to 10 °C
	$a_{2}=1.65\times 10^{-8}t^{2}+1.35\times 10^{-7}t-8.3\times 10^{-6}$	−10 °C to 10 °C
**10 hPa**	$a_{0}=-5.7126\times 10^{-4}t^{3}-0.0897t^{2}-4.2482t-61.84$	−70 °C to −30 °C
	$a_{1}=4.5028\times 10^{-5}t^{2}+2.0423\times 10^{-3}t+0.06285$	−70 °C to −30 °C
	$a_{2}=-1.175\times 10^{-8}t^{2}-6.415\times 10^{-7}t-1.1345\times 10^{-5}$	−70 °C to −40 °C
